# Decreased DACH1 expression in glomerulopathy is associated with disease progression and severity

**DOI:** 10.18632/oncotarget.13470

**Published:** 2016-11-19

**Authors:** Qing-Quan Liu, Ya-Qun Zhou, Hui-Quan Liu, Wen-Hui Qiu, Hui Liu, Ting-Yang Hu, Qing Xu, Yong-Man Lv, Kong-Ming Wu

**Affiliations:** ^1^ Department of Nephrology, Tongji Hospital, Tongji Medical College, Huazhong University of Science and Technology, Wuhan, China; ^2^ Department of Anesthesiology and Pain Medicine, Tongji Hospital, Tongji Medical College, Huazhong University of Science and Technology, Wuhan, China; ^3^ Department of Oncology, Tongji Hospital, Tongji Medical College, Huazhong University of Science and Technology, Wuhan, China

**Keywords:** DACH1, eGFR, glomerulopathy

## Abstract

Cell fate determination factor dachshund1 (DACH1) is a chromosome-associated protein that regulates cellular differentiation throughout development. Recent genome-wide association studies have show that missense mutation in *DACH1* leads to hereditary renal hypodysplasia. Renal DACH1 expression can be used to estimate glomerular filtration rate (eGFR). We firstly characterized the function of DACH1 in normal and diseased renal tissue using immunohistochemistry to assess DACH1 in human renal biopsy specimens from 40 immunoglobulin A nephropathy (IgAN) patients, 20 idiopathic membranous nephropathy (IMN) patients, and 15 minimal change disease (MCD) patients. We found that DACH1 expression was decreased in the nephropathy group relative to healthy controls. DACH1 staining in the glomerulus correlated positively with eGFR (r = 0.41, *p* < 0.001) but negatively with serum creatinine (r = −0.37, *p* < 0.01). *In vitro*, DACH1 overexpression in human podocytes or HK2 cells decreased expression of cyclin D1, but increased expression of p21 and p53, which suggested that DACH1 overexpression in human podocytes or HK2 cells increased the G1/S phase or G2/M cell arrest. Together, These findings indicate that DACH1 expression is decreased in glomerulopathy imply a potential role for DACH1 in the this development of human chornic glomerulopathy. These data suggest that DACH1 is a potential a marker of disease progression and severity for glomerular diseases.

## INTRODUCTION

The DACH1 protein is a key component of the retinal determination gene network (RDGN) family, originally found to be the primary regulator of *Drosophila* eye development, but now known to possess functions in tumorigenesis and organogenesis [1, 2]. Human *DACH1* maps to chromosome 13q21–q22 and encodes a nuclear transcription cofactor with two highly conserved domains that confer characteristic DNA-binding ability. DACH1 involves in cell differentiation and proliferation during renal developmental genes [3, 4]. The DACH1 protein is found in developing kidneys where epithelial/mesenchymal interactions are crucial in patterning and cell fate determination. DACH1 is also abundantly expressed in glomerular podocytes and tubular epithelial cells of the adult kidney [5]. Our previous study identified DACH1 as a candidate gene to cause hereditary renal hypodysplasia [6]. Deletion mapping has shown that *DACH1* is located in a chromosomal region related to congenital kidney anomaly [7]. In addition, DACH1 may be involved in the pathogenesis of branchio-oto-renal (BOR) syndrome, which comprises numerous congenital anomalies characterized by branchial arch deformation, hearing loss, and variable renal anomaly [8]. In the last several years studies have demonstrated that DACH1 expression level is associated with incident chronic kidney diseases and provides a means to estimate glomerular filtration rate (eGFR) [9–12].

Individuals with impaired kidney function, particularly those with glomerulopathy or renal tubulointerstitial diseases, are at increased risk for progression to chronic kidney disease (CKD), and even end stage renal disease (ESRD) [13, 14]. eGFR is the primary metric for CKD, which is defined as eGFR < 60 ml/min/1.73m2 [15]. We postulated that DACH1 expression may be altered in chronic kidney diseases and its function may be associated with the prognosis of renal diseases. Detailed cellular expression pattern and function of DACH1 in adult human kidney and the relationship between the expression of DACH1 and clinic-pathological characteristics in glomerular diseases have yet to be reported. The current study aims to clarify the immunological characteristics and the function of DACH1 protein associated with disease progression in glomerulopathy by means of immunohistology. In addition we show that DACH1 involved in regulating cell cycle–related proteins following ectopic. DACH1-transfected HK2 or human podocytes.

## RESULTS

### Baseline patient characteristics between groups

The clinical and biochemical characteristics of the control group and fifty patients with nephropathy are listed in Table 1. Analysis of patients’ clinical record revealed that age and gender were not significantly different between control and nephropathy groups (*p* > 0.05). The mean eGFR of the healthy control group was higher than that of any nephropathy group (*p* < 0.05), and the mean 24-hour urinary protein of the control group was dramatically lower than that of any nephropathy group (*p* < 0.05) at the time of sampling. However, no significant difference in clinical parameters mentioned above was observed among patients suffering from IgAN, IMN, and MCD. Additionally, no significant difference in plasma creatinine was recorded among these groups.

**Table 1:** Individuals’ clinical parameters at the time of renal biopsy

Characteristics	Normal	IgAN	IMN	MCD
	(*n* = 20)	(*n* = 40)	(*n* = 20)	(*n* = 15)
Age, years	45.3 ± 6.5	41.4 ± 6.7	45.3 ± 9.1	38.8 ± 9.5
Gender, Male/Female	8/12	19/21	9/11	7/8
Serum creatinine, umol/L	53.2 ± 14.6	74.6 ± 13.8	67.3 ± 15.1	69 ± 12.3
Urinary protein, g/day	0.15 ± 0.09	1.48 ± 0.62	2.95 ± 0.48	3.8 ± 0.93
eGFR,ml/min/1.1.73 m^-2^	126.8 ± 28.3	73.8 ± 33.4	73.5 ± 26.3	74.5 ± 30.2

Data expressed as mean ± SD, IgAN = immunoglobulin A nephropathy, IMN = idiopathic membranous nephropathy, MCD = minimal change disease, eGFR = estimated glomerular filtration rate.

### Characterization of DACH1 expression in renal tissues

**Figure 1:**
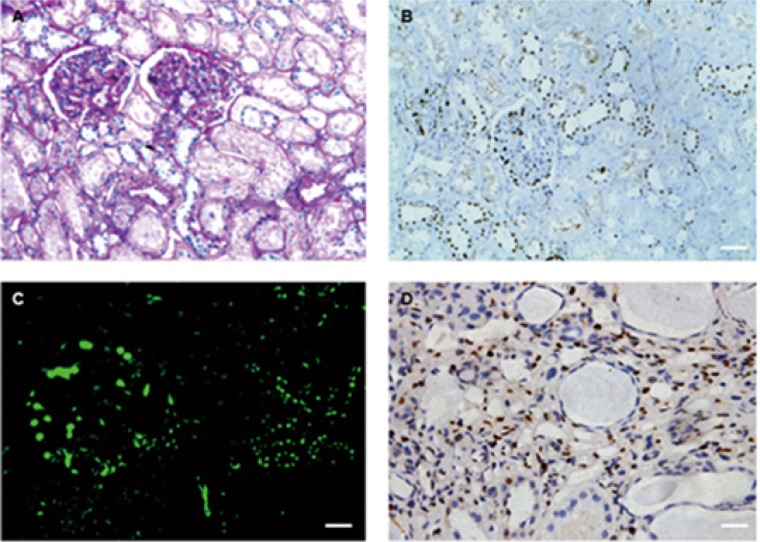
PAS staining, immunohistochemistry and immunofluorescence for DACH1 (**A**) PAS staining in normal kidney. (**B**) Sequential sections with A and showing positive staining for DACH1 in human normal kidney. (**C**) Immunofluorescence for DACH1, showing the intensity of expression of DACH1 in glomerular is higher than tubules. (**D**) DACH1 staining in renal tissue with fibrosis, seeing renal interstitium was positive for DACH1. Original magnification × 400, Bar, 50 μm.

We performed immunohistochemical examination on normal renal tissues from nephrectomy specimens. In all cases, DACH1 staining appeared primarily in glomerulus and renal tubules. Interestingly, the intensity of DACH1 staining was higher in glomerulus than in tubules. DACH1 is predominantly expressed in distal tubular epithelial cells (identified by their morphological features of cuboidal epithelium with little brush border and open tubular lumens) and located mainly in the nucleus (Figure 1A, 1C). For initial characterization of DACH1 staining in diseased kidney tissues, we examined renal biopsies from glomerular disease. We observed DACH1 positive staining in glomerular and distal tubular epithelial cells. DACH1 staining occasionally was found in renal interstitium of diseased samples with glomerulopathy accompanied by chronic renal lesion, but was absent from the renal interstitium of normal tissue (Figure 1B, 1D).

### Kidney biopsy tissue immunohistochemistry analysis

**Figure 2:**
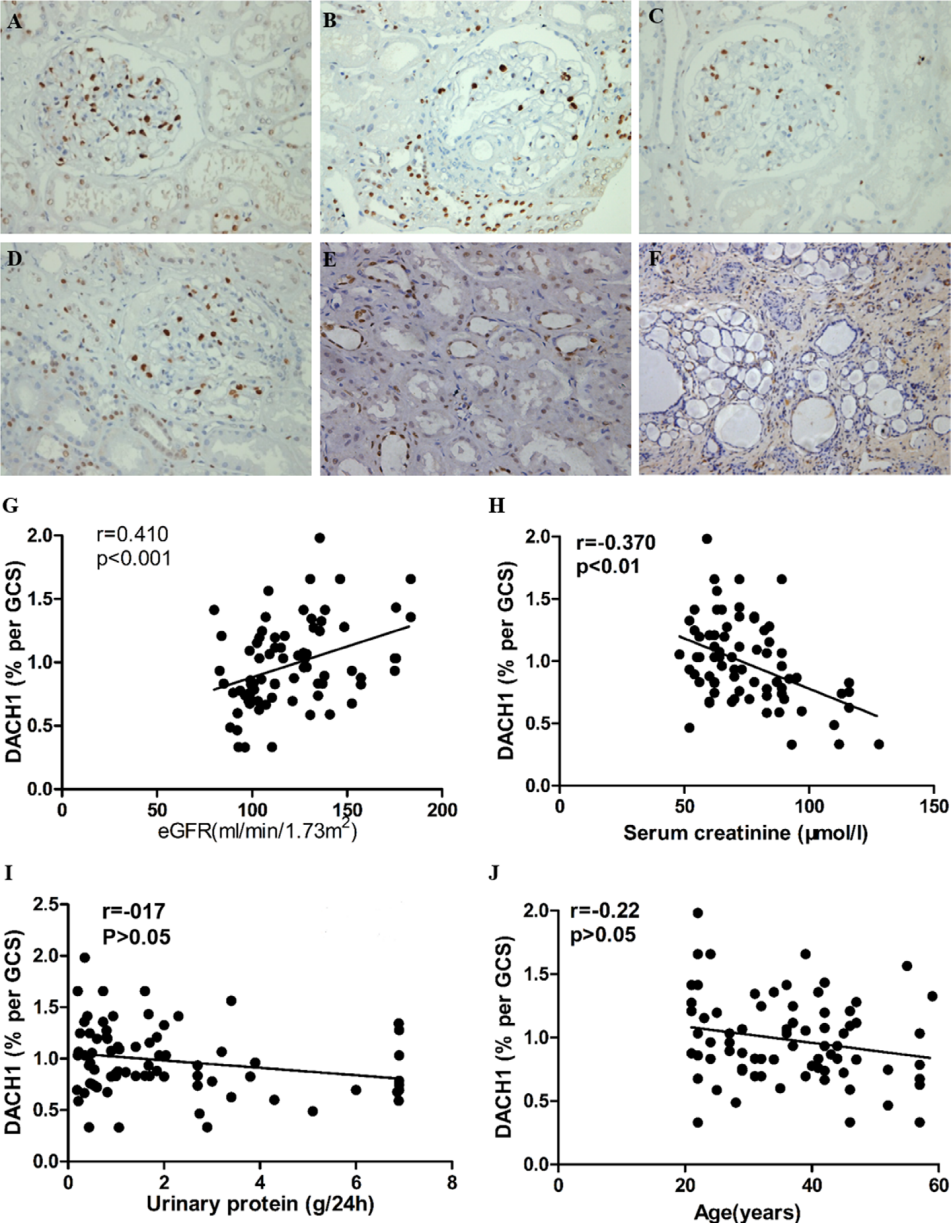
DACH1 expressed in normal and glomerulopathy renal tissue, and correlated with clinical parameters (**A**) DACH1 staining in normal renal tissue obtained from nephrectomy specimens. (**B**, **C**, and **D**) DACH1 staining for minimal change disease, immunoglobulin A nephropathy, and idiopathic membranous nephropathy, respectively. (**E**) DACH1 expression in normal kidney tubules. (**F**) DACH1 staining in lesioned renal tissue. The DACH1 expression in glomerulus shows a positive correlation (**G**) with eGFR and a negative correlation (**H**) with serum creatinine. Such level reveals no relevance with 24 h urinary protein (**I**) and patients’ age. All photos shown display immunoperoxidase stains with hematoxylin counterstain. All tissue sections are counter-stained with periodic acid-Schiff; original magnification × 400, Bar, 50 μm.

**Figure 3:**
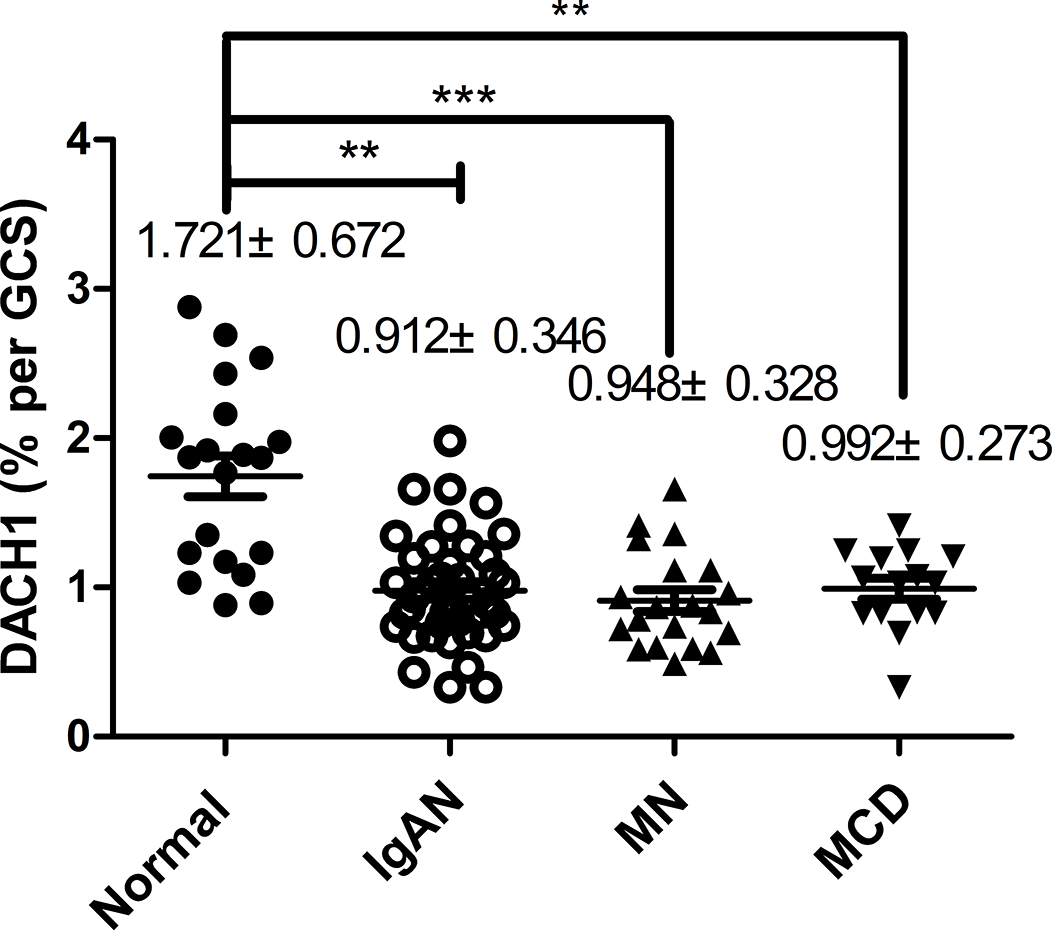
The magnitude of DACH1-expressing in glomerulus Compared with normal kidneys, DACH1 expression was decreased in kidneys associated with IgAN, MN, and MCD, **P < 0.001, ***P < 0.01, **P < 0.01, respectively. IgAN vs MN, IgAN vs MCD, MN vs MCD, *P* > 0.05, respectively, Tukey’s Multiple comparison test. The horizontal bars represent medians.

We performed immunohistochemistry (IHC) for DACH1 on renal biopsies with a range of primary glomerular lesions. There was more positive DACH1 immunostaining in normal renal glomerulus and kidney tubules than in nephropathy tissues (*p* < 0.05) (Table 2) and more severe renal pathological lesions were associated with less glomerular and tubular DACH1 expression (Figure 2A–2F). Moreover, DACH1 expression in lesioned and dilated renal tubules was substantially lower than that in normal kidney tubules (Figure 2E, 2F). Further analysis of DACH1 staining in renal glomerulus among normal kidney tissue and different types of glomerulopathy tissues showed that normal cells exhibited the highest DACH1 expression (*p* < 0.001). However, there was no significant statistical difference among the IgAN, IMN, and MCD samples (*p* > 0.05) (Figure 3). Collectively, these results suggest that DACH1 expression was down-regulated at times of renal lesion.

**Table 2:** DACH1 immunostaining level in glomerulus (glom) and renal tubules (rb) in both diseased and normal samples

Group	DACH1 (glom0	DACH1 (rb)
Nephropathy (*n* = 75)	0.0098 ± 0.00038^a^	0.00734 ± 0.00027^b^
Normal (*n* = 20)	0.0164 ± 0.0013	0.0132 ± 0.00075

Data expressed as mean ± SEM, ^a^*P* < 0.01, ^b^*P* < 0.01, glomerulopathy group vs normal group.

### Correlations between DACH1 expression in renal tissue and patients’ clinical biochemical profiles

The correlation between renal DACH1 expression and clinical parameters at the time of biopsy was analyzed. Glomerular cell DACH1 staining intensity was positively correlated with estimated glomerular filtration rate (eGFR, r = 0.41, *p* < 0.001) (Figure 2G), and showed a robust negative association with serum creatinine (r = −0.37, *p* < 0.01) (Figure 2H). However, the 24-hour urinary protein (r = −0.17, *p* > 0.05) and patients’ age (r =0.22, *p* > 0.05) at the time of biopsy were not clearly associated with glomerular DACH1 expression (Figure 2I, 2J). Notably, we found that DACH1 expression levels tended to be negatively related with 24-hour urinary protein in those patients who developed renal function failure, although the difference did not reach statistical significance. In normal cases, no evident DACH1 staining was observed in renal interstitium, but in nephropathy tissues with obvious renal lesions, intermittent staining was shown in the area.

### Correlation of DACH1 expression with disease progression and parameters predicting clinical outcome

**Figure 4:**
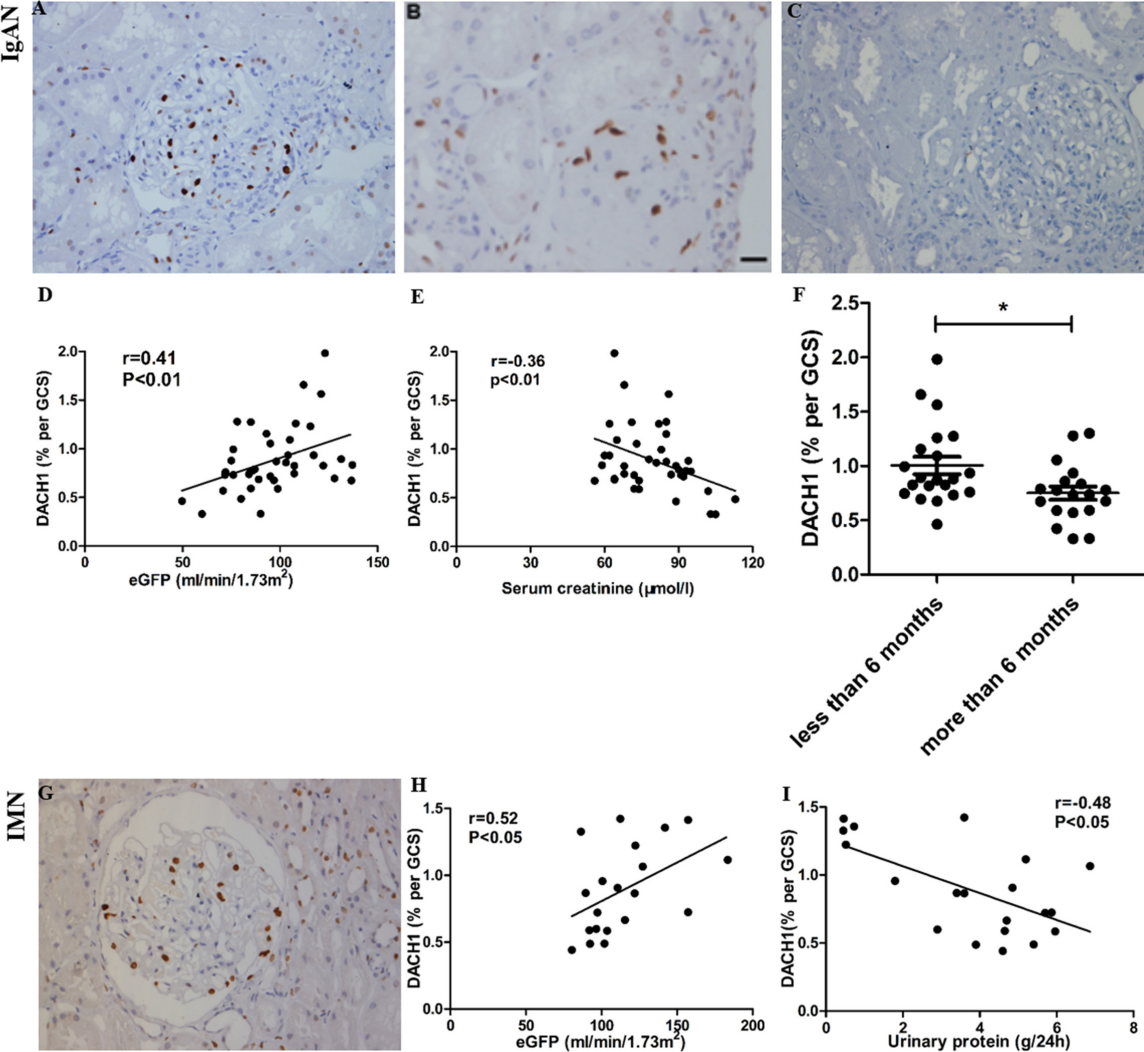
DACH1 expression in immunoglobulin A nephropathy (IgAN) and idiopathic membranous nephropathy (IMN) and its correlation with clinical parameters (**A** and **C**) positive and negative control (NC) stained for DACH1 in a focal proliferative IgAN. (**B**) A modicum glomerular staining for DACH1 in scarred glomerulus in sclerotic class IgAN. (**D**) Glomerular DACH1 expression correlates strongly with eGFR in IgAN. (**E**) Glomerular DACH1 expression correlates negatively with serum creatinine in IgAN. (**F**) DACH1 detection is higher in patients with disease duration of less than 6 months. (**G**) section with positive staining for DACH1 in a phase II IMN. (**H**) Glomerular DACH1 expression correlates strongly with eGFR in IMN. (**I**) Glomerular DACH1 expression correlates negatively with 24 h urinary protein in IMN. All photos shown contain immunoperoxidase stains with hematoxylin counterstain, 400 magnification. GCS, glomerular cross section, Bar, 50 μm.

Among all patients with IgAN, the mean glomerular DACH1 protein level of those who suffered the disease for less than 6 months was significantly higher than that in those who suffered from the disease for a longer period (0.88% DACH1 per GCS for a duration of less than 6 months versus 0.74% per GCS for a duration of more than 6 months; *P* = 0.019). In addition, glomerular DACH1 expression was positively correlated with eGFR (r = 0.41; *P* < 0.01) and negatively associated with serum creatinine (r = −0.36, *P* < 0.01; Figure 4A–4F).

In IMN, glomerular DACH1 expression also correlated with eGFR at the time of biopsy (r = 0.52; *P* < 0.05). We found that glomerular DACH1 expression negatively correlated with the level of 24 hours urinary protein (r = −0.48; *P* < 0.05; Figure 4G–4I). These characteristics suggest that DACH1 expression is an indication of disease severity. Among all patients with MCD, no correlation between DACH1 expression with serum creatinine or proteinuria was discovered.

### DACH1 appears to regulate apoptosis and cell proliferation

**Figure 5:**
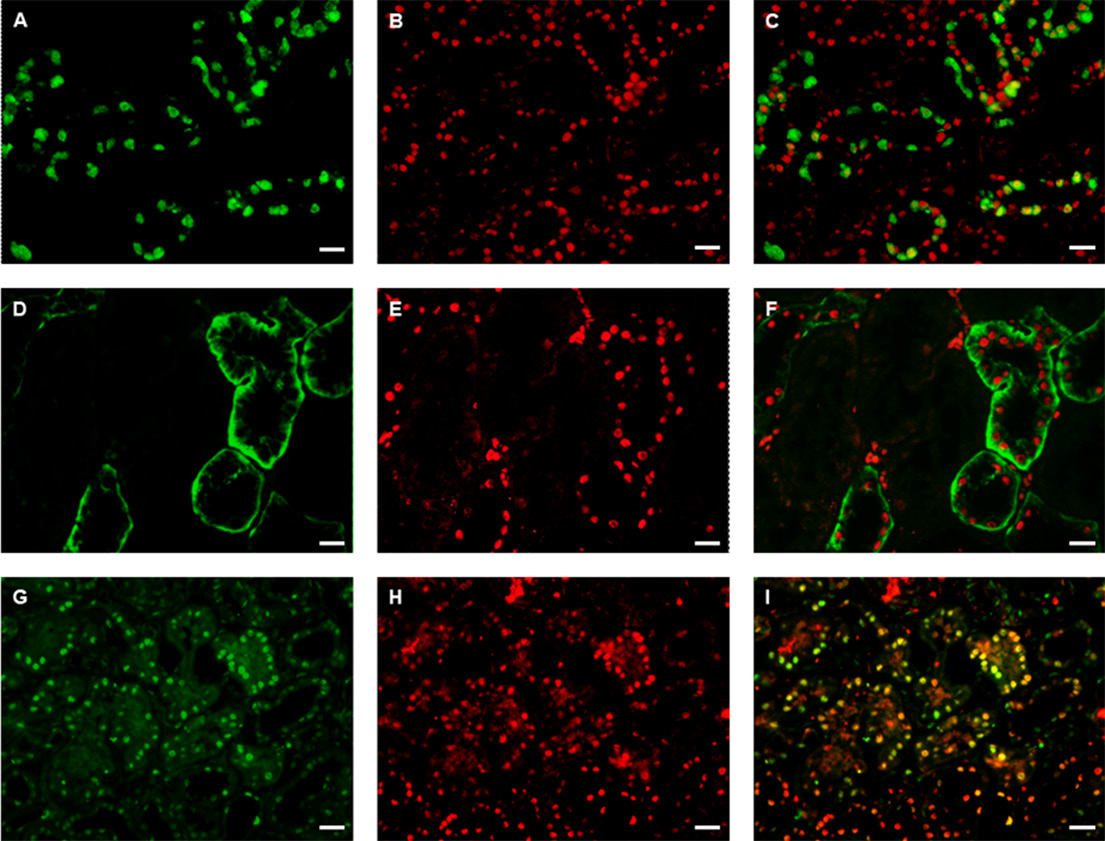
Double immunofluorescence staining for DACH1 and Bax/Bcl-2, PCNA in normal kidney A representative micrograph of double immunofluorescence was performed for DACH1 (red) (**B**, **E**, **H**), Bcl-2 (green) (**A**), Bax(green) (**D**), PCNA (green) (**G**), Merged images (**C**, **E**, **I**). Original magnification is × 400, Bar, 50 μm.

We performed staining for DACH1, Bax, Bcl-2, and PCNA on sections of renal tissues (representative images shown in Figure 5A–5I). This immunofluorescence staining showed that DACH1 appeared to localize to areas expressing Bax and Bcl-2, which are proteins involved in regulating cell apoptosis. This is consistent with another finding where samples with severe apoptosis pathology demonstrated the lowest DACH1 expression among all biopsies. Interestingly, double immunofluorescence staining revealed strong co-localization of DACH1 and PCNA. These results indirectly imply that DACH1 might participate in regulation of cell proliferation and apoptosis.

### DACH1 and downstream cell cycle-related proteins in renal tissues

**Figure 6:**
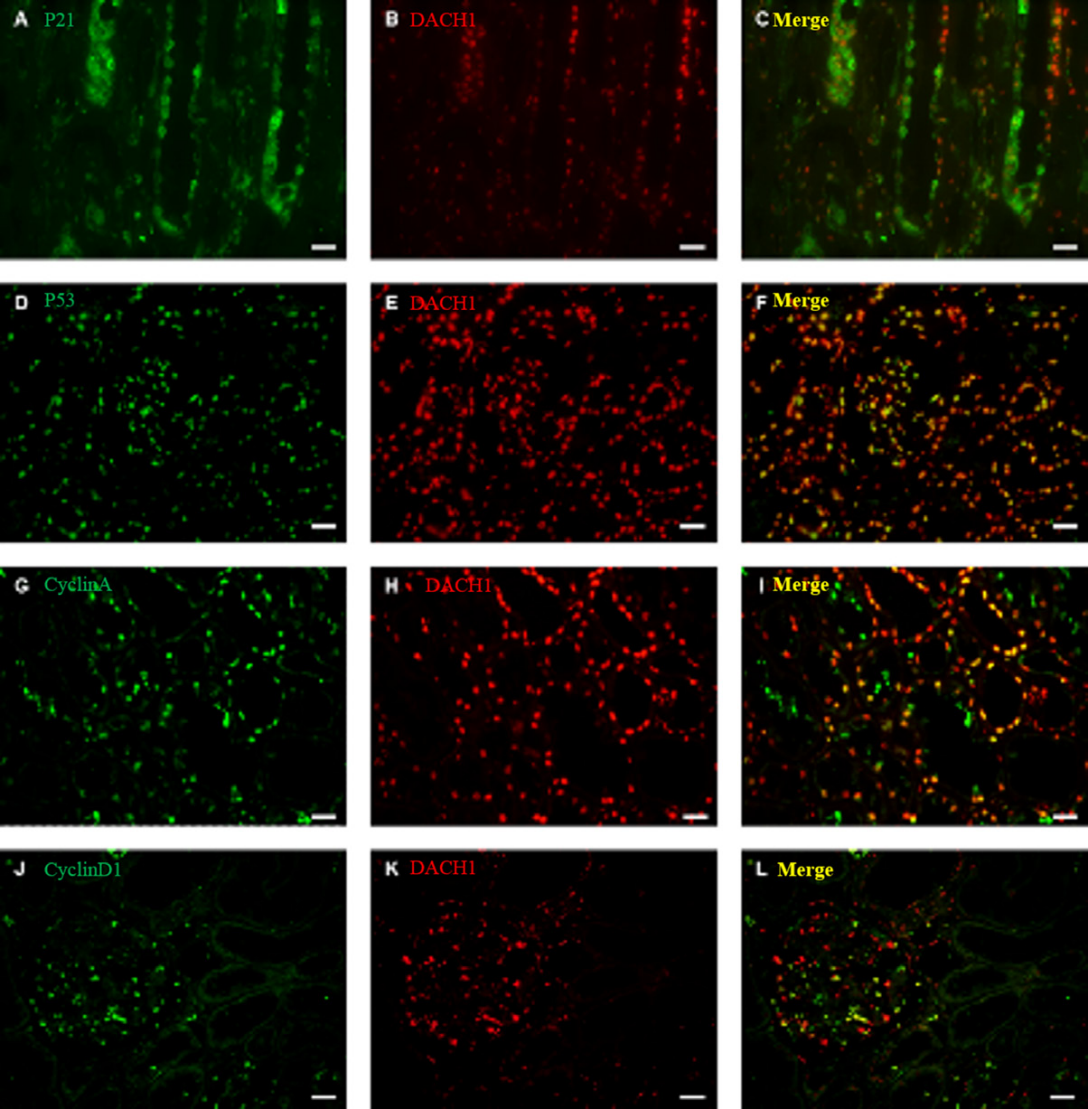
Double immunofluorescence staining for DACH1 and P21, P53, Cyclin A, Cyclin D1 in normal kidney A representative micrograph of double immunofluorescence was performed for DACH1(red) (**B**, **E**, **H**, **K**), P21 (green) (**A**), P53(green) (**D**), Cyclin A (green) (**G**), Cyclin D1 (green) (**J**), Merge images (yellow) (**C**, **F**, **I**, **L**). Original magnification is ×400, Bar, 50 μm.

To further clarify the possible mechanism of DACH1 in regulating apoptosis and cell proliferation, we performed double-labeling immunofluorescence for DACH1 and p21, p53, cyclin D1, and cyclin A in sections of renal tissues (representative images shown in Figure 6A–6L). p21 expression weakened obviously when DACH1 was co-expressed in same cells (Figure 6A–6C). In addition, double immunofluorescence staining revealed co-localization of DACH1 and p53 proteins. When DACH1 was positive expression in some cells, then these cells expression of p53 enhanced obviously, indicating that DACH1 protein might interact with p53 and participate in regulating cell cycle (Figure 6D–6F). The majority of DACH1+ cells expressed cyclin A or cyclin D1 (Figure 6G–6L), which suggests that DACH1 might regulate cyclin A or cyclin D1 expression. Together these results suggest that DACH1 participates in regulation of the expression of proteins involved in cell cycle, subsequent inhibition of DNA synthesis, and cellular proliferation.

### Expression of DACH1and cell cycle-related proteins were changed during temperature-switched cell growth arrest in podocytes

The human podocytes maintain cell proliferation at the permissive temperature of 33°C, and only pomote cell differentiation at the nonpermissive temperature of 37°C. To assess the possible change of DACH1 proteins in podocyte growth arrest, human podocytes cells were respectively maintained at 33°C and 37°C for 10 days. As shown in Supplementary Figure S1A, western blotting analysis indicated that the temperature switch to 37°C induced an approximate 1.8-fold increase in DACH1 protein expression compared with at 33°C. Furthermore, we found that cell cycle regulator p21 expression was upregulated, but the CylinD1 and PCNA expression were downregulated (Supplementary Figure S1A, S1B). These observations speculate that DACH1 maybe regulate podocyte proliferation by targeting p21 and CylinD1.

### Effects of DACH1 overexpression on cell cycle-related proteins *in vitro*

**Figure 7:**
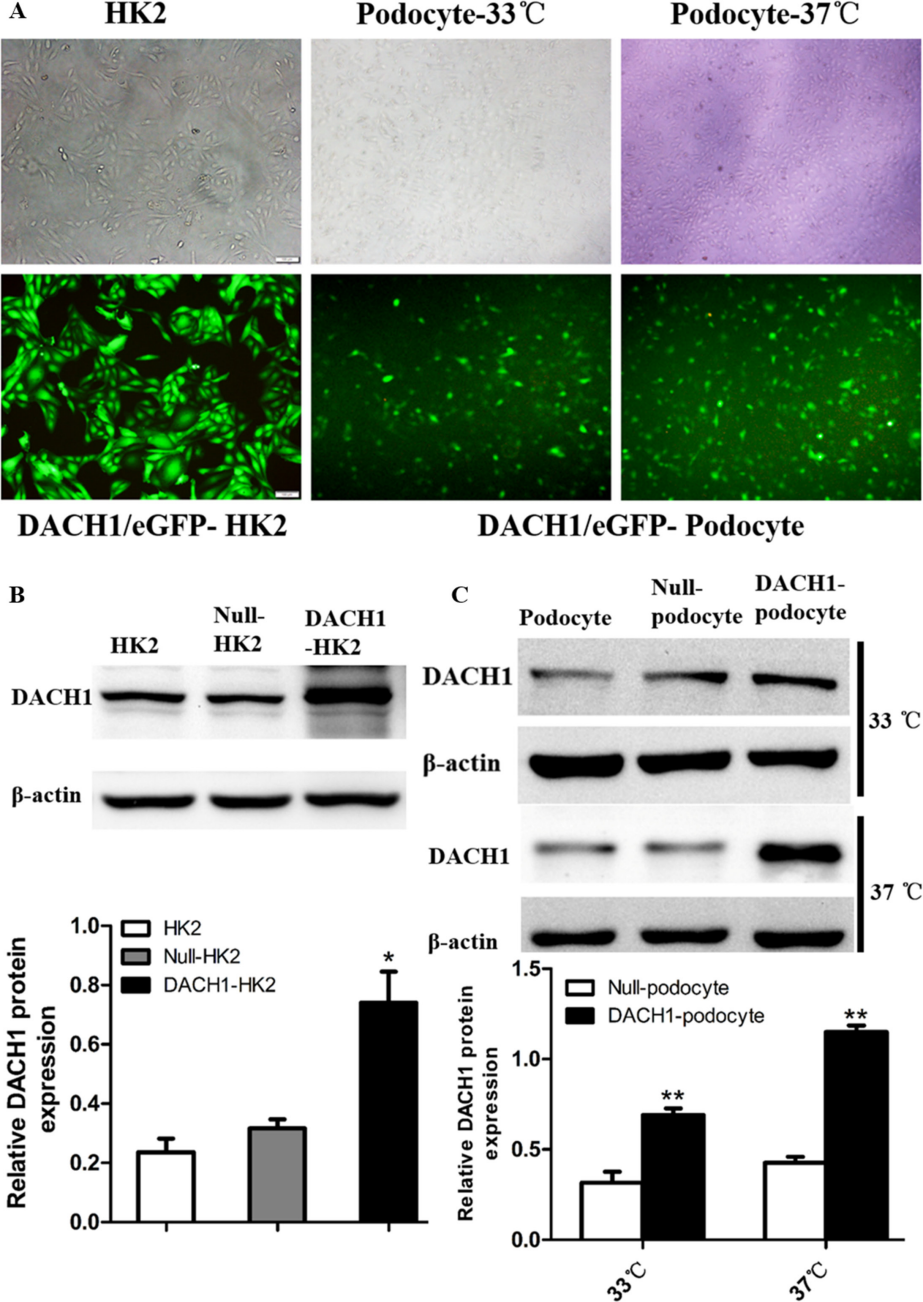
HK2 and human podocytes transfected with the DACH1 plasmid (**A**) HK2, podocyte cultured in a permissive temperature (33°C) and cultured in a nonpermissive temperature (37°C). transfection with Plasmid.GFP. (**B**) Western blotting analysis of the DACH1 protein in Plasmid. DACH1-transfected HK2 and summarized data showing that DACH1 expression. (**C**) Western blotting analysis of the DACH1 protein in Plasmid. DACH1-transfected podocytes and summarized data showing that DACH1 expression. The data are displayed as mean ± SEM; *P < 0.05; **P < 0.01 vs. Null-HK2 or Null-podocyte. All experiment were performed at least thrice with samples from independent experiments.

**Figure 8:**
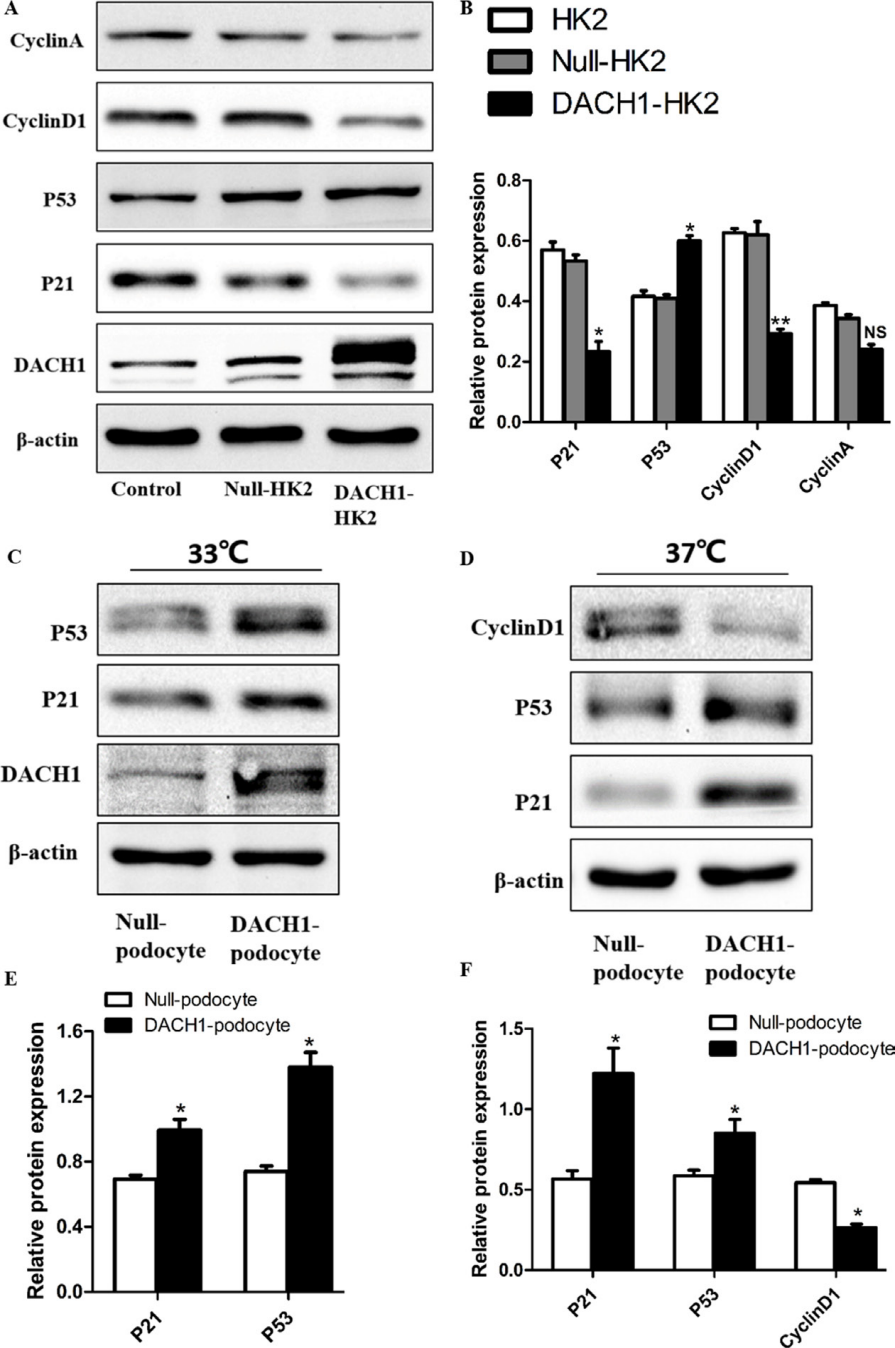
Effects of DACH1 overexpression on cell cycle-related proteins in HK2 and Podocytes (**A**) Representative Western blot images and (**B**) summarized data showing DACH1, p21, p53, CyclinD1, CyclinA expression with Plasmid. DACH1-transfected HK2. (**C**) representative Western blot images and (**E**) summarized data showing that the abundance DACH1, p21, p53 were increased in Plasmid. DACH1-transfected podocytes cultured in a permissive temperature (33°C). (**D**) representative Western blot images and (**F**) summarized data showing that the abundance p21, p53 were increased, while CyclinD1 was decreased in Plasmid. DACH1-transfected podocytes cultured in a permissive temperature (37°C). *P < 0.05 vs. Null-HK2 or Null-podocyte. All experiment were performed at least thrice with samples from independent experiments.

To further decipher DACH1’s effect on the cell cycle in HK2 cells, we overexpressed DACH1 in human podocytes and HK2 cells (human proximal tubular cells) and examined changes in downstream gene expression and biological features of the cells. At 48 hours post-infection with DACH1 or control plasmid, DACH1 was significantly upregulated by immunofluorescence and western blotting analysis (Figure 7A–7C). The expression of cyclin D1 was significantly down-regulated in DACH1-overexpressing HK2 cells than that in control plasmid groups (Figure 8A–8B). A more dramatic increase of DACH1 resulted in p53 and p21 expression was found in plasmid DACH1 group, but the changes in cyclin A expression were not significantly different in plasmid DACH1 group, a slight decrease was found comparing with in the controls (*P* = 0.06) (Figure 8A–8B). In addition, DACH1 significantly increased CyclinB1 protein expression, and the the ration CyclinD1 to CyclinB1 was remarkably decreased (Supplementary Figure S2A).

We, also, further detected the cell cycle-related proteins change of podocytes under the infections the plasmid of DACH1. The expression of p53 and p21 was significantly up-regulated in DACH1-overexpressing human podocytes than that in control plasmid groups at the permissive temperature of 33°C (Figure 8C, 8E). Furthermore, the expression of PCNA was significantly decreased when podocytes overexpressed DACH1 in the condition of 33°C (Supplementary Figure S2B). Next, when podocytes were cultured at the nonpermissive temperature of 37°C and infection with DACH1 or control plasmid, western blotting analysis revealed that the expression of p53 and p21 elevated in DACH1-overexpressing group, in contrast, the cyclin D1 protein significantly decreased (Figure 8D, 8F). Thus, these data collectively showed that exogeneous expression of DACH1 in HK2 and podocytes could significantly limit the cell proliferation through induction of regulating the cell cycle progression.

## DISCUSSION

Our study, involving 60 patients, is the first systematic analysis of DACH1 expression from the perspective of renal disease. We have found that the level of glomerular DACH1, which was expressed in some distal tubule cells and podocytes according to immunohistochemical study, is an indicator of renal function insufficiency. The frequency of DACH1 expression in tubules and podocytes was decreased in damaged kidney, but tended to increase in renal interstitium. We deduced that DACH1 expression correlates with disease progression, and that this protein might be involved in regulating cell proliferation and apoptosis in renal tissue with pathological lesions. To further confirm the regulation of podocyte and HK2 cells cycle progression by DACH1, western blotting analysis revealed that p21 and p53 protein was upregulated, the CyclinD1 was downregulated after plasmid DACH1-infected podocyte and HK2 cells. Taken together, These results showed that DACH1 plays a role in disease pathogenesis and exogenous expression of DACH1 alone could involve in regullating cell cycle-related proteins.

Across the entire cohort, DACH1 expression levels correlated with serum creatinine and eGFP at the time of biopsy, and with the accepted severity of different glomerular pathologies in IgAN. Moreover, glomerular DACH1 expression in those who suffered from the disease for less than 6 months was significantly higher than that in those suffered for a longer period. Among all patients with IMN, expression of the protein, apart from the positive relation with eGFR, was negatively correlated with 24 h urinary albumin excretion (UAE), a valuable risk marker for renal complications and a measurement long considered to be the prime diagnostic test to assess early severity of nephropathy [16,17]. This discovery is consistent with previous reports that DACH1 is associated with estimated glomerular filtration rate (eGFR) and CKD by genome-wide association studies [10–12,18]. All of these findings suggest that DACH1 is a disease progression marker of renal function failure. Glomerular DACH1 expression levels were the highest in normal renal tissue, obviously higher than in the IgAN, IMN, and MCD, the three most common renal histopathologic patterns among the entire spectrum of primary glomerular diseases [19, 20]. No difference in glomerular DACH1 expression was detected among the IgAN, MN, and MCD groups, which indicates that its level is not influenced by type of glomerular disease. Taken together, it can be deduced that DACH1 is a potential marker of disease severity and disease progression in glomerulopathy.

Exploring the mechanism of DACH1 in human disease has, to date, mostly focused on its role in restricting tumor cell proliferation and inhibiting solid tumor migration and invasion due to DACH1 down-regulation in cancer tissues [21, 22]. *DACH1* and other *RDGN*-related genes are associated with mammalian renal development. In the 16-dpc mouse kidney, the DACH1 protein was detected in developing nephrons, including many of the convoluted tubules, most of mesenchymal cells, “comma-shaped” bodies, and podocytes of the glomeruli, but the collecting ducts did not express DACH1[5]. *DACH1* is a candidate gene for branchio-oculo-facial syndrome and Fraser syndrome based on the phenotypic presentation [5]. Our previous study found that *DACH1* mutation enhanced suppression of the TGF-β/BMP4 pathway and could act synergistically in promoting the development of renal hypodysplasia [6]. We observed significant DACH1 colocalization with Bax and PCNA, which suggested that DACH1 may be involved in regulating cell proliferation and apoptosis. *In vitro*, post-infected with plasmid DACH1 or podocytes expression DACH1 increased, podocytes expression of PCNA protein was significantly decreased. This is consistent with our previous work, which reported that DACH1 level is inversely correlated with PCNA expression and that it inhibits cellular proliferation in kidney cancer [23]. Additionally, a recent study claimed that cytoplasmic and nuclear expression of DACH1 involved in cell proliferation in osteosarcoma; for instance, cytoplasmic DACH1 might promote cell proliferation [24].

DACH1 has been shown to involve in regulating cell-cycle and be associated with proliferation in most of tumour cell. Kalousova et al reported that DACH1-mediated repression of p27Kip1was associated with perinatal pancreatic β cell proliferation[25]. Thus, it could be involved in regulating cell-cycle and proliferation of DACH1-infected podocytes and HK2 in the similar ways. In this study, it was found that DACH1 positive cells also expressed cell cycle related proteins, such as p21, p53, cyclin D1, and cyclin A, in renal tissues by immunofluorescence double staining. In vitro, HK2 cells overexpressed with DACH1 resulted in p21 and p53 upregulated, but the cyclin D1 and cyclin A downregulated, and the ratio of cyclin D1/cyclin B1 was decreased. As we known, the major role of cyclin D1 promote G1–S phase transition [26]. p53 activation initiates cell cycle arrest and proximal tubular epithelial cells (PTC) apoptosis [27]. It is well known that cyclin A, cyclin B1, and the CDK inhibitor p21 involved in the regulation of G2/M transition [28]. Megyesi et al. demonstrated that upregulation of p21 attenuated CDDP-induced tubular damage or against ischemia/reperfusion injury [29]. Therefore, these data suggest that the expression of DACH1 in damaged tubular cells may be mediate cell cycle arrest at G1/S phase or G2/M phase arrest, which contribute to PTC repair. These findings were consistent with our previous work, which demonstrated that DACH1 represses p21^CIP1^ and S-phase requires p53-mediated activity [30, 31]. Our previous study also demonstrated that DACH1 inhibited cyclin D1 expression and inversely correlated with PCNA in renal cancer cells [23] or blocked cell proliferation through a c-Jun DNA-binding partner [32]. It is consistant with our previously study that DACH1 inhibited c-Jun induction of cyclin A expression [33].

The mature podocyte is a terminally special differentiated epithelial cell that serves specialized functions in the glomerulus, mature podocytes exit the cell cycle and cease proliferating so that podocytes exhibit a quiescent phenotype [34]. But studies have shown that podocytes undergo DNA synthesis and mitosis, but no cytokinesis in some of glomerular diseases [35]. These lead to binucleated or multinucleated podocytes. In the model of mesangioproliferative nephritis (mesangioproliferative nephropathy (anti-Thy 1.1 nephritis model), the number of cyclin D1-positive podocytes was increased significantly [36]. In the model of membranous nephropathy report that Cdc2, cyclin B1, and cyclin B2 were increased in podocytes despite absent proliferation [35]. In the present study, we found a marked decrease in DACH1 protein expression in human nephropathy, especially in glomerulus. Herein, we initially studied the role of the *DACH1* gene in adult kidney diseases outside of the tumor field. During temperature-switched growth arrest of huamn podocytes, DACH1 and p21 expression were significantly upregulated, while cyclin D1 was significantly downregulated, which sugggested the the change of DACH1 expression was associated with cell cycle. Furthermore, *in vitro* experiments were performed to assess the effect of DACH1 on podocytes cell cycle regulation and proliferation. We found that overexpression of DACH1 resulted in upregulating p53, p21, while decreasing PCNA expression in the condition of the permissive temperature of 33°C. Differentiated podocytes overexpression of DACH1 significantly enhanced p21 and p53 level, however, decreased cyclin D1 expression compared with control podocytes. These results suggested that DACH1 was significant repression of G1/S phase progression of the cell cycle. Likewise, several other reports have shown that in podocytes, the up-regulation of p21 and p53 expression is closely related to G2/M phase arrest and the subsequent inhibition of proliferation [37]. Studies have shown that p53 and p53-dependent increases in p21 were required to halt cell cycle progression to allow adequate time for DNA repair mechanisms following DNA damage [38]. Accordingly, we speculate that the DACH1 may contribute to DNA repair by inhibit podocyte proliferation at G_1_/S and G_2_/M phases.

However, this study had some limitations, such as lack of multivariate analysis to correct confounding factors. The number of cases used in this study was, however, insufficient to allow us to perform more specific analysis. The patients for kidney biopsy was intentionally selected by taking into account their serum creatinine, a primary risk factor for bleeding complication after transcutaneous kidney biopsy [39–41]. Transcutaneous kidney biopsy was not considered for patients whose serum creatinine exceeded 90 μmol/L, which is why nephropathy was not complicated with severe renal lesion in this study. Therefore, DACH1 expression showed no dramatic reduction in glomerulus and renal tubules. Yet, it was interesting to note that there was statistical difference in mean DACH1 between the normal group and the diseased groups. We will explore the relevant mechanism in detail to elucidate the function of DACH1 protein in human kidney in a future study.

In conclusion, the results of our study suggest that DACH1 expression is decreased in glomerulopathy and involved in regulating cell cycle-related proteins imply a potential role for DACH1 in the this development of human chornic glomerulopathy. Toghter, these data suggest that DACH1 is a potential a marker of disease progression and severity for glomerular diseases.

## MATERIALS AND METHODS

### Patients and sample collection

From the September 2014 to December 2014, a total of 75 nephropathy patients were selected. The tissues were from renal biopsy performed for diagnostic purposes in Tongji Hospital. Control tissues were obtained from 20 patients who underwent nephrectomy for renal neoplasms, from sites remote from renal cell carcinoma-bearing tissue. These patients, renal function is normal and eGFR > 90 ml.min^−1^.1.73 m^−2^. Paraffin-embedded, formalin-fixed tissues were used for histopathological examination and immunohistochemical staining. These specimens were classified as morphologically “normal” human kidney samples. Our study was approved by the ethics committee of the Huazhong University of Science and Technology, Tongji Hospital. The disease samples investigated included: IgAN (*n* = 40), IMN (*n* = 20), and MCD (*n* = 15). Clinical data corresponding to the renal specimens were collected and presented as mean and standard deviation (Table 1). The eGFR was estimated according to the Modification of Diet in Renal Disease (MDRD) equation: eGFR (ml.min^−1^.1.73 m^−2^) = 186 × plasma Cr (mg/dl)^−1.154^ × age (years)^−0.203^ (× 0.742, if female subjects), where the Cr indicates serum creatinine [14, 42].

### Renal tissue immunohistochemistry

Paraffin-embedded specimens were serially sectioned into 3 μm thick sections, and mounted on polylysine-coated histology slides. The immunohistochemistry protocol was performed according to manufacturer’s instructions. Briefly, sections were dewaxed in xylene, and dehydrated in different concentrations of ethanol. Endogenous peroxidase activity was quenched by incubation of slides with 3% H_2_O_2_ solution. Samples were blocked with 5% normal blocking serum and then incubated with primary antibody overnight at 4°C. The primary antibody is polyclonal rabbit anti-human specific for DACH1 (1:300 dilution, Proteintech, Wuhan, China). After washing in phosphate-buffered saline, the slides were incubated with goat anti-rabbit IgG antibody conjugated to horseradish peroxidase (DAKO, Tokyo, Japan) for 30 min at room temperature. The slides were then counter-stained with hematoxylin stains. Negative controls were established by replacing primary antibody with PBS.

### Renal tissue immunofluorescence

For immunofluorescence double staining, paraffin-embedded specimens were stained using a modified indirect immunofluorescence method. Primary antibodies were selected against DACH1 (Proteintech, Wuhan), Cyclin D1, Cyclin A, p21, p53, PCNA, Bcl-2 and Bax (Santa Cruz Biotechnology, Dallas, TX). DyLight 488and Cy3-conjugated secondary antibodies (Jackson Immuno Research, West Grove, PA) were then used. Colocalization was analyzed by immunofluorescence microscopy.

### Image analysis

The mean optical density of DACH1 immunohistochemical images were analyzed in the glomerulus or renal tubules by Image-Pro Plus software (Version 6.0); the mean optical density of each field was estimated by the tissue IOD values per unit area of each measured field. An independent examiner in a blind study randomly selected ten serial non-overlapping high power fields (400×) per glomerulus and renal tubule (barring glomerulus) for DACH1 staining from each slide imaged by light microscopy (Olympus BX-50), and the results were taken as an average of selected fields.

### Cell culture

Conditionally immortalized human podocytes were cultured as described previously [43]. Briefly, human podocytes were conditionally immortalized by introducing a temperature sensitive SV40-T antigen by transfection. These cells proliferate in growth medium containing RPMI 1640 (Gibco,USA) supplemented with 10% fetal bovine serum (Gibco, USA), 1× penicillin-streptomycin, 1 mM L-glutamine and 1 × ITS (Invitrogen, Grand Island, NY, USA) at a permissive temperature (33°C). When the podocytes reached approximately 70–80% confluence, they were transferred to 37°C for differentiation in a medium without insulin, transferrin, or selenium (ITS) for approximately 10–14 days.

Human kidney-2 (HK2) cells, which are immortalized human renal proximal tubular epithelial cells, were purchased from American Type Culture Collection (ATCC, USA), and cultured in DMEM/F12 (Gibco, Carlsbad, CA) medium supplemented with 10% FBS (Gibco, Australia), 100 U/ml penicillin, and 100 μg/ml streptomycin in a humidified atmosphere containing 5% CO2 at 37°C.

### Transfection

The human DACH1 plasmid and empty vectors were purchased from GeneCopoeia (catalog no. EX-Z8223-M98, Rockville, MD, USA). The DACH1 plasmid or empty vectors (EX-NEG-M98, Rockville, MD, USA) were transfected into podocytes and HK-2 cells using Lipofectamine 2000 reagent (Invitrogen, USA), according to the manufacturer’s instructions. Briefly, 3 × 10^5^ podocytes or 1 × 10^5^ HK-2 cells were seeded in 6-well plates, 12–24 h later, the cells were transfected with complexes consisting of 2 ug DACH1 plasmid or a negative control. The podocytes and HK-2 cells were incubated with the transfection complexes under normal growth conditions for 48 h, and then, the cells were collected for western blot analysis.

### Western blotting

Podocyte proteins were extracting by lysing cells in RIPA buffer (Beyotime, Haimen, China) with protease/phosphatase inhibitors and then centrifuging at 12,000 g × 30 min at 4°C. Protein concentration of each sample was measured by BCA reagent kit, and protein samples were mixed with 5 × loading buffer and boiled for 8 min at 98°C. Then, cellular protein (20–40 μg) was separated by SDS-PAGE gels electrophoresis, electro-transferred to PVDF membranes (Millipore Corporation, Billerica, MA, USA). DACH1 rabbit polyclonal antibody (1:1000 dilution, Proteintech, Wuhan, China), Cyclin D1, PCNA mouse monoclonal antibody (1:1000 dilution, Santa Cruz Biotechnology, Dallas, TX), Cyclin A, Cyclin B1, p21, p53 rabbit polyclonal antibody (1:500, Affinity Biosciences, USA) were used as primary antibodies. Western blots were probed with anti-mouse or goat HRP-conjugated secondary antibodies (1:5000, Jackson, USA) for 1 hour at 37°C. Protein bands were detected using an enhanced chemiluminescence detection system (Bio-Rad, USA). β-actin or β-tubulin was used as an internal control. Band intensity was quantified using ImageJ software (1.44 P).

### Statistical analysis

Data corresponding to the renal samples are presented as mean ± SEM. Differences between two groups in continuous variables were analyzed by Mann-Whitney *U* test. Association of categorical variables was examined by the chi-square test. Multiple comparisons among groups were analyzed using a one-way ANOVA or the Kruskal-Wallis test. The correlations between clinical parameters and DACH1 expression were evaluated by Spearman’s nonparametric correlation coefficient test. P values < 0.05 were considered to represent a statistically significant difference and all tests were two-tailed. All statistical analyses were performed using SPSS, version 16.0 software (SPSS, Chicago, IL) and GraphPad Prism version 6.0 software (Graph software, San Diego, CA).

## SUPPLEMENTARY MATERIALS FIGURES


