# Supplementary material for: Decreased DACH1 expression in glomerulopathy is associated with disease progression and severity
Source: Oncotarget. 2016 Nov 19;7(52):86547–60. doi: 10.18632/oncotarget.13470 (PMC5349934; doi:10.18632/oncotarget.13470)
Supplement: Supplementary file 1 [file oncotarget-07-86547-s001.pdf]

Decreased DACH1 expression in glomerulopathy is associated with disease progression and severity

Supplementary Materials

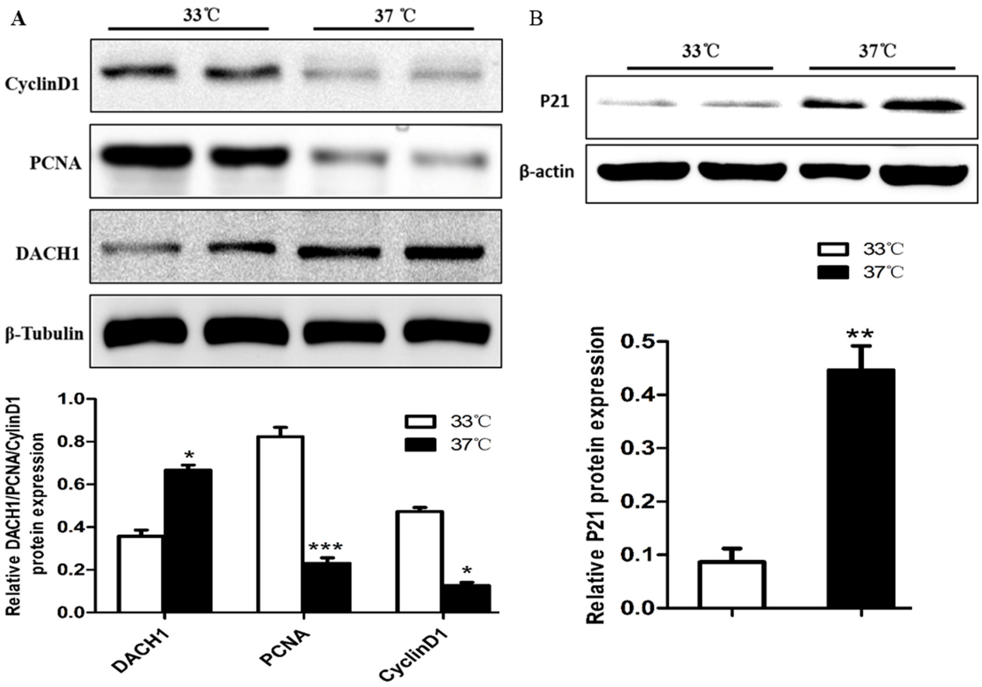

Supplementary Figure S1: Expression of DACH1and cell cycle-related proteins were changed during temperature-switched cell growth arrest in podocytes. (**A**) Representative Western blot images and summarized data showing DACH1 was upregulated, while PCNA, CyclinD1 were downregulated during podocytes cultured by temperature-switched. (**B**) representative Western blot images and summarized data showing that p21 was increased during podocytes cultured by temperature-switched. $*P < 0.05$, $**P < 0.01$, $***P < 0.001$ vs. cultured in a permissive temperature (33°C). All experiment were performed at least thrice with samples from independent experiments.

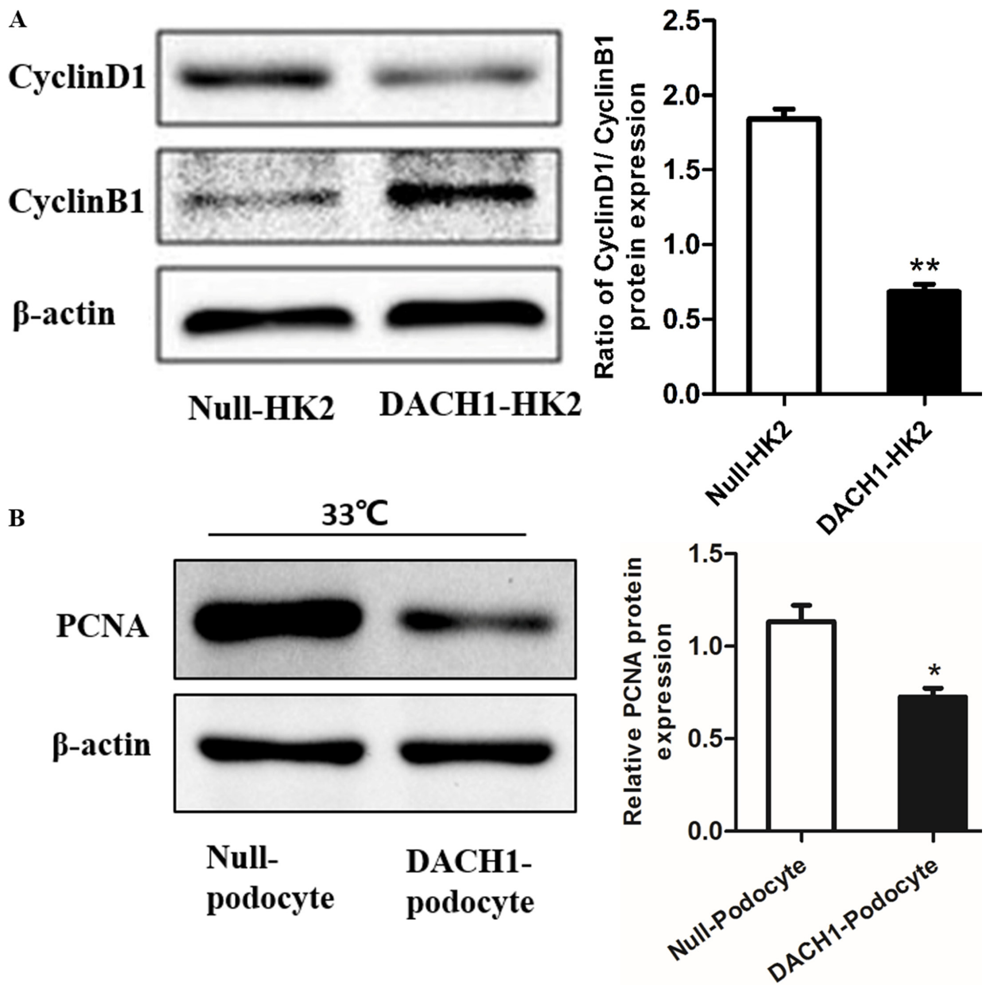

Supplementary Figure S2: Effects of DACH1 overexpression on cell cycle-related proteins in HK2 and Podocytes. (**A**) Representative Western blot images showing cyclin D1 and cyclin B1 expression and summarized data showing the raito of cyclin D1/ cyclin B1 after Plasmid.DACH1-transfected HK2. (**B**) representative Western blot images and summarized data showing that the abundance PCNA was decreased in Plasmid.DACH1-transfected podocytes cultured in a permissive temperature (33°C). **P* < 0.05,***P* < 0.01 vs. Null-HK2 or Null-podocyte. All experiment were performed at least thrice with samples from independent experiments.
